# Caregiving-Related Depression Increases Neuroinflammation in Spousal Caregivers to Individuals With Cognitive Impairment: A Longitudinal Study

**DOI:** 10.1093/gerona/glae235

**Published:** 2024-09-19

**Authors:** So Yeon Jeon, Hee Won Yang, Bo Ran Son, Jimin Baek, Jeong Lan Kim

**Affiliations:** Department of Psychiatry, Chungnam National University Hospital, Daejeon, Republic of Korea; Department of Psychiatry, College of Medicine, Chungnam National University, Daejeon, Republic of Korea; Department of Psychiatry, Chungnam National University Hospital, Daejeon, Republic of Korea; Brain Research Institute, College of Medicine, Chungnam National University, Daejeon, Republic of Korea; Department of Psychiatry, Chungnam National University Hospital, Daejeon, Republic of Korea; Department of Psychiatry, Chungnam National University Hospital, Daejeon, Republic of Korea; Department of Psychiatry, College of Medicine, Chungnam National University, Daejeon, Republic of Korea

**Keywords:** Caregiving burden, Dementia, Glial fibrillary acidic protein, Neurofilament light chain, Psychological stress

## Abstract

**Background:**

The caregiving burden of the spousal caregivers (SCGs) to individuals with cognitive impairment poses public health challenges with adverse psychosocial and physiological effects. However, few studies have investigated the neurobiological impact of caregiving, particularly through the investigation of neuroinflammation and neurodegeneration.

**Methods:**

Using data from a longitudinal cohort at Chungnam National University Hospital, the relationship between caregiving burden, neuroinflammation, and neurodegeneration was examined in 38 older adult couples over a 16-month period. Caregiving burden was assessed through a multifaceted approach. For factors related to the care recipient, we assessed cognitive function and neuropsychiatric symptoms. Factors regarding the SCGs included the measurement of perceived depression. Glial fibrillary acidic protein (GFAP) was used as a plasma biomarker for neuroinflammation and neurofilament light chain (NfL) for neurodegeneration. Regression analyses were adjusted for age, sex, apolipoprotein E status, follow-up interval, vascular risk factors, and physical activity.

**Results:**

Changes in depression among SCGs were significantly correlated with increased GFAP levels (*p* = .003), indicating that greater depressive symptoms during caregiving are associated with increased neuroinflammation. In contrast, no significant correlations were found between changes in cognitive function or neuropsychiatric symptoms in care recipients and the plasma biomarker levels of SCGs. Additionally, there was no significant association between changes in depression and NfL levels in SCGs.

**Conclusions:**

The psychological stress experienced by SCGs while caring for partners with cognitive impairment actively contributes to neuroinflammation, a well-known risk factor for various diseases. This study emphasizes the need to address psychological stress experienced by older adult caregivers.

Caregiving for older adults with cognitive decline, which has been identified as a prototypic chronic stressor (1), constitutes a significant public health challenge with well-documented adverse psychosocial and physiological ramifications (2–5). The clinical manifestations experienced by caregivers include depressive symptoms (6–8), social isolation (9), and decreased physical activity (10). These manifestations not only compromise the caregiver’s quality of life but also correlate with increased risks of cognitive impairment (7,11–14) and cardiovascular diseases (11,12,15) in caregivers. These adverse impacts are especially harmful to spousal caregivers (SCGs), as they are typically older adults simultaneously undergoing a natural decline in their immune system (16). Longitudinal analyses have demonstrated that compared with their non-caregiving counterparts, SCGs face a 63% increased relative risk of all-cause mortality (17), implying profound health implications associated with caregiving for SCGs.

The link between caregiving burden and adverse health outcomes is partly attributed to the inflammatory process, as evidenced by elevated levels of C-reactive protein (CRP) and cytokines such as interleukin-6, and tumor necrosis factor-alpha, along with reduced antibody levels (3,18–20). The overproduction of these cytokines and inflammatory markers is associated with age-related health problems, including cardiovascular disease (21) and all-cause dementia (22,23). In a 2-year longitudinal study, an increased CRP levels were linked to the functional decline of caregivers to individuals with dementia but not to non-caregivers (20). Existing evidence further supports the connection between chronic psychological stress and neuroinflammatory and neurodegenerative processes (24). Moreover, a longitudinal study with a 14-year follow-up demonstrated that SCGs have an increased risk of all-cause dementia, specifically with a 1.6-fold increase (13). Another longitudinal study reported that, even after the death of the care recipient, SCGs to individuals with dementia showed consistently accelerated cognitive decline compared with SCGs to individuals without dementia (14). Despite these findings, the direct impact of caregiving burden on neuronal damage, specifically neuroinflammation and neurodegeneration, remains underexplored. Recent evidence strongly suggests that the glial fibrillary acidic protein (GFAP) and neurofilament light chain (NfL) are indicators of ongoing neuroinflammation and neurodegeneration (25,26). GFAP, which is indicative of astrocytic activation (27), and NfL (28), which is a marker of axonal damage, both provide valuable insights into the pathological processes underlying neurological disorders. Notably, recent large-scale longitudinal studies have reported that elevated plasma GFAP and NfL levels are strongly correlated with an increased risk of all-cause dementia (25,29–32). This suggests that caregiving-related stress could be linked to neuroinflammation and neurodegeneration. However, the relationship between caregiving burden and these plasma biomarkers in SCGs to individuals with cognitive impairment is yet to be determined.

Therefore, this study investigated the relationship between the caregiving burden in caregivers to individuals with cognitive impairment and plasma levels of GFAP and NfL in SCGs. The caregiving burden was evaluated using a multifaceted approach to investigate the factors influencing the neurobiological impact among SCGs. We assessed perceived depression among SCGs for factors relating to the SCGs. Additionally, given the significant correlation between the severity of cognitive impairment and neuropsychiatric problems in care recipients and the caregiving burden (33,34), we assessed cognitive function and neuropsychiatric symptoms for factors related to the care recipient.

## Method

### Study Design and Participants

This study was part of a longitudinal cohort study focusing on older adult couples engaged in spousal caregiving at the Chungnam National University Geriatric Psychiatry Clinic. Enrollment in the cohort began in May 2020, and annual assessments were conducted. The analysis included older adult couples registered up to May 2023 and selected those who had completed 2 rounds of evaluation. The detailed methods are described in our previous study (35). At every assessment, the measurement of cognitive function and neuropsychiatric symptoms of the care recipients was performed along with clinical evaluations of the SCGs, which included assessments of depression and cognitive function and blood tests. To measure the cognitive functions of both care recipients and SCGs, we utilized the Korean version of the Mini-Mental State Examination (MMSE) (36). This study analyzed the data from 38 couples who completed all baseline and follow-up evaluations. Of the SCGs, 23 cared for individuals with dementia and 15 cared for those without dementia. The inclusion criteria for the SCGs were as follows: (1) age between 55 and 90 years, (2) serving as the primary caregiver for a spouse, (3) ability to live independently, (4) not diagnosed with dementia, and (5) not diagnosed with major depressive disorder at enrollment. Dementia was diagnosed based on the DSM-IV criteria established by the American Psychiatric Association in 1994. This study was approved by the Ethical Review Committee of Chungnam National University Hospital (approval number 2020-05-002). Informed consent was obtained from all participants prior to their involvement.

### Clinical Assessments for Caregiving Burden

During the study, the SCGs underwent detailed clinical evaluations conducted annually by a skilled neuropsychological technician and a research nurse. The caregiving burden was evaluated based not only on the perceived depression of the SCGs but also on the cognition and neuropsychiatric symptoms of the care recipient.

Depressive symptoms in the SCGs were measured using the Korean version of the Geriatric Depression Scale (GDS) (37). GDS scores of 16 or above are considered to be an indication for major depression screening (37). We evaluated the severity of cognitive impairment in care recipients assessed by the MMSE and neuropsychiatric symptoms, which are established factors associated with caregiving burden in SCGs (4,33).

To assess neuropsychiatric symptoms in care recipients, we used the 12-item neuropsychiatric inventory (NPI) (38). This inventory spans a range of neuropsychiatric symptoms across 12 domains: delusions, hallucinations, agitation (or aggression), depression/dysphoria, anxiety, elation (or euphoria), apathy (or indifference), disinhibition, irritability (or lability), aberrant motor behavior, nighttime disturbances, and changes in appetite. Upon endorsement of any symptoms, SCGs were prompted to evaluate both the frequency and intensity of the symptoms within each domain. These evaluations were used to calculate a score for each domain (with a maximum of 12 points). Any domain not endorsed by a caregiver was assigned a score of 0. The sum of the domain scores yielded the total NPI score for each care recipient, with a maximum score of 144 points.

### Assessment for Covariates

Comorbid vascular risk factors in SCGs, including diabetes mellitus, hyperlipidemia, hypertension, coronary artery disease, transient ischemic attack, and stroke were assessed based on ongoing pharmacological treatments or medical histories. This assessment was conducted through comprehensive interviews and corroborated by a trained nurse, forming the basis for the Vascular Risk Score (VRS), which represents the cumulative presence of vascular risk factors as a percentage (39). Body mass index (BMI) was calculated by dividing the participants’ weight in kilograms by the square of their height in meters, with measurements taken and calculated by research nurses. The Korean version of the International Physical Activity Questionnaire (IPAQ) was used to assess physical activity levels in participants (40). This questionnaire comprises 7 items that record the total time (in minutes) spent on moderate to vigorous physical activities, including walking and periods of inactivity, over the past 7 days. Responses were converted into metabolic equivalent task (MET) minutes per week (MET-min/week) following the IPAQ scoring guidelines (41). An average MET score was calculated for each type of activity using the following MET values: walking = 3.3 METs, moderate physical activity = 4.0 METs, and vigorous physical activity = 8.0 METs. The overall physical activity level was determined by summing the MET-min/week values from walking, moderate activity, and vigorous activity.

### Blood Sampling and Laboratory Assessments

Genomic DNA was isolated from whole-blood samples, and apolipoprotein E (APOE) genotyping was conducted as previously described (42). Presence of the APOE ε4 allele (APOE4) was confirmed if at least one ε4 allele was identified. Levels of plasma GFAP and NfL were measured through the Single Molecule Array (SIMOA) method using the Simoa Neurology 2-Plex A assay kit (Quanterix, Billerica, MA) and DNA Link (DNA Link Inc., Seoul, Republic of Korea) to carry out the immunoassay procedures. In a standard experimental setup, both samples and controls were allocated to 96-well Quanterix plates for duplicate analysis, including a 4× dilution performed by the device, following the manufacturer’s instructions. The assay employed a SIMOA HD-X machine (Quanterix) for a 2-step immunoassay where target antibodies on paramagnetic beads were mixed with the sample and a biotinylated detection antibody in a single step. This allows the target molecules in the sample to be simultaneously captured by the bead-coated antibodies and detected using the biotinylated antibody. The minimum detectable limit was 0.241 pg/mL for NfL and 0.467 pg/mL for GFAP, as determined analytically. Data acquisition was performed using a SIMOA HD-X analyzer operated with SIMOA HD-X software (Quanterix), version 3.1.2011.30002.

### Statistical Analysis

We compared SCGs to individuals with and without dementia to assess potential differences in caregiving burden and its impact on plasma biomarkers. This comparison aimed to explore whether clinically relevant differences exist in SCG variables based on the diagnostic status of the care recipient.

And, we conducted linear regression analyses to explore the association between the caregiving burden and the levels of GFAP and NfL among SCGs at baseline. In Model 1, the age and sex of the SCGs were adjusted as covariates. Model 2 expanded upon Model 1 by including APOE4 status, VRS and IPAQ scores as additional covariates, along with BMI, for GFAP analysis. In the longitudinal analysis, changes between baseline and follow-up assessments were quantified by calculating the differences in variables, followed by linear regression analyses to further examine these relationships. This involved analyzing the connection between alterations in the GDS scores of SCGs and the MMSE (or NPI) scores of care recipients and the corresponding changes in GFAP and NfL levels. Moreover, the longitudinal analyses were adjusted for the interval between assessments and the baseline values of the GDS scores of SCGs and MMSE (or NPI) scores of care recipients. Bonferroni correction was applied for multiple comparisons using *p* < .05/2 (=number for each dependent variable [GFAP and NfL]).

To explore the moderating effects of sex, APOE4 status, and clinical diagnosis on caregiving burden and plasma biomarkers, variables that showed associations with plasma biomarkers with a statistical significance of *p* < .025 were selected for further analyses. Multiple linear regression analyses were conducted after controlling for the age of the SCGs, MMSE scores of the care recipients, APOE4 status, VRS, IPAQ scores and other selected factors. These analyses included interaction terms (eg, the MMSE score of the care recipient multiplied by sex) as independent variables, with the chosen factor serving as the dependent variable.

In an exploratory manner, we analyzed the relationship between plasma biomarkers and MMSE scores in SCGs, both cross-sectionally and longitudinally. Initially, comparisons were made between baseline plasma biomarker levels and MMSE scores of the SCGs. We explored whether baseline plasma biomarker levels and changes in these biomarkers were associated with changes in the MMSE scores of the SCGs. Given the exploratory nature of this analysis, adjustments were made solely for age, sex, and APOE4 status, including the interval between assessments in the longitudinal analyses. All analyses were performed using SPSS 21 software (SPSS Inc., Chicago, IL), and *p* values < 0.05 (2-sided) were considered to indicate statistical significance.

## Results

### Characteristics of the Study Population

Table 1 shows the demographic and clinical characteristics of the study population at baseline. In the whole sample, the mean baseline age was 72.9 years (standard deviation, 6.1 years). Age was significantly associated with NfL (*r* = 0.448, *p* = .005), but showed trend level association with GFAP (*r* = 0.297, *p* = .074). Among care recipients, 23 (60.5%) were diagnosed with dementia. The SCGs to individuals with dementia were slightly older than those to individuals without dementia, although this difference was not significant. The BMI of SCGs to individuals with dementia was lower than that of SCGs to individuals without dementia. However, no significant differences were observed between the 2 groups in terms of sex, educational level, or type of care provided (Table 1).

**Table 1.** Characteristics of Study Population

Variables	SCGs to Individuals With Dementia (*n* = 15)	SCGs to Individuals Without Dementia (*n* = 23)	Total (*N* = 38)	*p* Value
SCG factors				
Demographic variables				
Age (years)	70.8 ± 5.3	74.3 ± 6.3	72.9 ± 6.1	.084
Female sex, *n* (%)	8 (53.3%)	14 (60.9%)	22 (57.9%)	.901
Education years	8.5 ± 3.8	8.5 ± 4.8	8.5 ± 4.4	.970
Household, *n* (%)				.677
without other family members	10 (66.7%)	18 (78.3%)	28 (73.7%)	
with other family members	5 (33.3%)	5 (21.7%)	10 (26.3%)	
Types of care, caring alone (%)	15 (100.0%)	23 (100.0%)	38 (100.0%)	
Clinical variables				
APOE4 carrier, *n* (%)	4 (26.7%)	3 (13.0%)	7 (18.4%)	.528
VRS (%)	15.6 ± 18.3	22.5 ± 17.8	19.7 ± 18.1	.256
Diabetes mellitus, *n* (%)	2 (13.3%)	7 (30.4%)	9 (23.7%)	.411
Hypertension, *n* (%)	5 (33.3%)	11 (47.8%)	16 (42.1%)	.583
Hyperlipidemia, *n* (%)	6 (40.0%)	10 (43.5%)	16 (42.1%)	1
Stroke, *n* (%)	0 (0.0%)	1 (4.3%)	1 (2.6%)	1
TIA, *n* (%)	15 (100.0%)	23 (100.0%)	38 (100.0%)	1
CAD, *n* (%)	1 (6.7%)	2 (8.7%)	3 (7.9%)	1
Body mass index (kg/m^2^)	25.7 ± 2.0	23.7 ± 3.7	24.5 ± 3.3	.035
IPAQ (MET × min/week)	2123 ± 2671	2082 ± 2541	2098 ± 2558	.962
Baseline GDS	4.0 ± 2.8	5.0 ± 3.1	4.6 ± 3.0	.304
MMSE	25.5 ± 2.7	25.4 ± 3.3	25.4 ± 3.0	.890
Follow-up interval (months)	14.9 ± 4.5	16.9 ± 4.2	16.1 ± 4.4	.163
Plasma biomarker				
Baseline GFAP (pg/mL)	165.2 ± 83.9	203.1 ± 68.1	188.2 ± 76.0	.134
Baseline NfL (pg/mL)	18.9 ± 9.1	23.3 ± 13.9	21.6 ± 12.3	.287
Care recipient factors				
MMSE	24.5 ± 2.9	16.4 ± 4.8	19.5 ± 5.8	<.001
Global CDR				<.001
0	3 (20.0%)	0 (0.0%)	3 (7.9%)	
0.5	12 (80.0%)	6 (26.1%)	18 (47.4%)	
1	0 (0.0%)	12 (52.2%)	12 (31.6%)	
2	0 (0.0%)	5 (21.7%)	5 (13.2%)	
NPI	15.3 ± 11.8	26.3 ± 20.9	21.8 ± 18.4	.074

*Notes*: Data are presented as mean ± *SD* or *n* (%). APOE = apolipoprotein E; CAD = coronary artery disease; CDR = Clinical Dementia Rating; GDS = Geriatric Depression Scale; GFAP = glial fibrillary acidic protein; IPAQ = International Physical Activity Questionnaire; MET = metabolic equivalent task; MMSE = Mini-Mental State Examination; NfL = neurofilament light chain; NPI = Neuropsychiatric Inventory; SCG = spousal caregiver; *SD* = standard deviation; TIA = transient ischemic attack; VRS = vascular risk factor score.

### Baseline Association of Caregiving Burden With GFAP and NfL Levels Among SCGs

At baseline, no significant correlation was found between the GDS scores and levels of GFAP (β = 0.076, *p* = .687) and NfL (β = −0.025, *p* = .612) among SCGs. Similarly, the MMSE or NPI scores of care recipients showed no association with GFAP (β = 0.261, *p* = .170 for MMSE scores; β = −0.073, *p* = .696 for NPI scores) and NfL levels (β = 0.006, *p* = .973 for MMSE scores; β = −.059, *p* = .727 for NPI scores) in SCGs (Table 2). Further, no significant associations were found between the NPI subdomains and GFAP and NfL levels (Supplementary Table 1).

**Table 2.** Baseline Association of Caregiving Burden With GFAP and NfL Levels Among SCGs

	Baseline GFAP			Baseline NfL		
	β	*t*	*p* Value	β	*t*	*p* Value
Model 1*						
Baseline GDS of SCG	0.069	0.399	.693	−0.038	−0.231	.819
Baseline MMSE of care recipient	0.214	1.242	.223	0.005	0.033	.974
Baseline NPI	−0.160	−0.241	.811	−0.026	−0.251	.804
Model 2†						
Baseline GDS of SCG	0.076	0.408	.687	−0.025	−0.150	.612
Baseline MMSE of care recipient	0.261	1.408	.170	0.006	0.035	.973
Baseline NPI	−0.073	−0.394	.696	−0.059	−0.353	.727

*Notes*: APOE = apolipoprotein E; BMI = body mass index; GDS; Geriatric Depression Scale; GFAP; glial fibrillary acidic protein; IPAQ = International Physical Activity Questionnaire; MMSE = Mini-Mental State Examination; NfL; neurofilament light chain; NPI = neuropsychiatric inventory; SCG = spousal caregiver; VRS = vascular risk factor score.

*Model 1: Adjusted for age and sex of SCGs.

†Model 2: Adjusted for age, sex, APOE4 postivity, VRS, IPAQ scores of SCGs (and BMI for GFAP analyses).

### Longitudinal Association of Caregiving Burden With GFAP and NfL Levels Among SCGs

**Figure 1.**
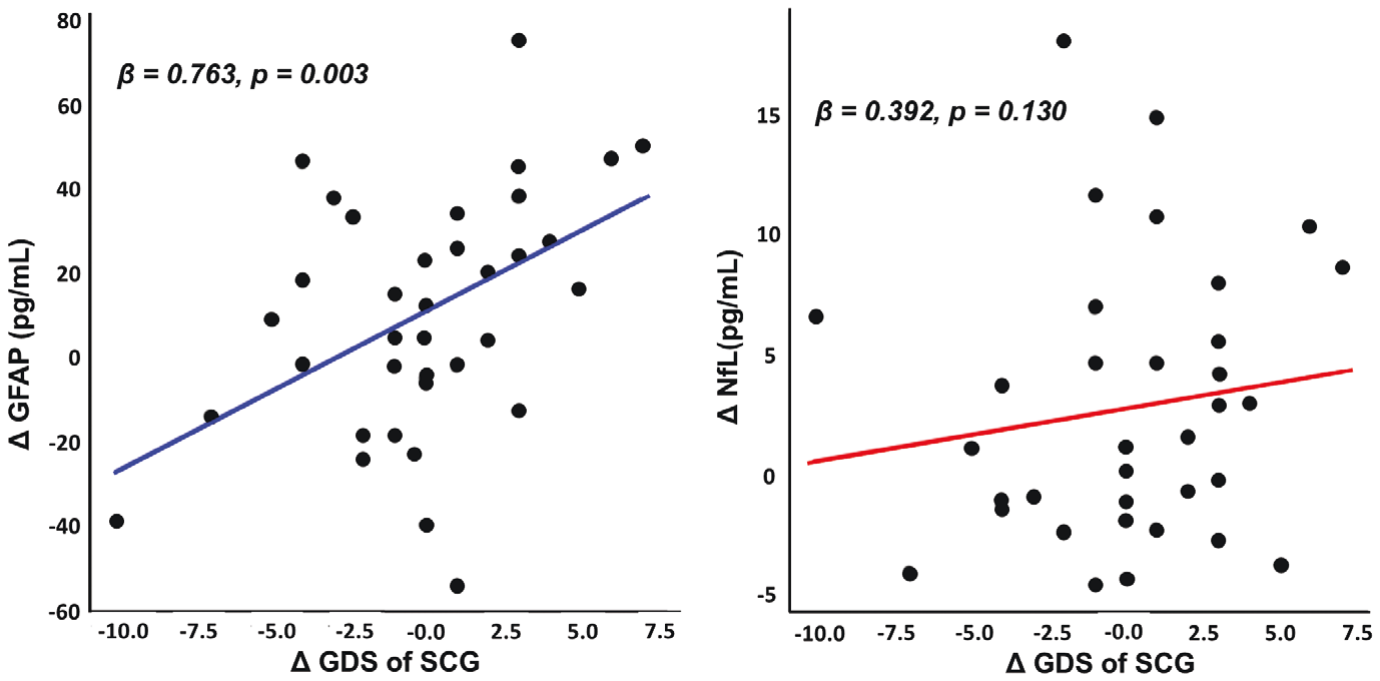
Longitudinal association of changes in depression and changes in (**A**) GFAP and (**B**) NfL among SCGs. Multiple linear regression analyses were performed after controlling for age, sex, APOE4 status, VRS, IPAQ scores, follow-up interval, and baseline GDS of the SCGs (and BMI for GFAP analyses). APOE = apolipoprotein E; BMI = body mass index; GDS = Geriatric Depression Scale; GFAP = glial fibrillary acidic protein; IPAQ = International Physical Activity Questionnaire; MMSE = Mini-Mental State Examination; NfL, neurofilament light chain; SCG = spousal caregiver; VRS = vascular risk factor score.

Changes in GDS among SCGs were significantly correlated with changes in GFAP levels, a relationship that persisted even after adjusting for VRS, BMI, IPAQ scores, APOE4 status, and baseline GDS (β = 0.763, *p* = .003; Table 3 and Figure 1). This indicates that an increase in depressive symptoms among the SCGs during the evaluation period was associated with a corresponding increase in GFAP levels. No significant correlation was observed between changes in GDS and NfL levels (β = 0.392, *p* = .130). Changes in MMSE or NPI scores for care recipients did not show a significant relationship with changes in GFAP (β = 0.087, *p* = .705 for MMSE scores; β = −0.188, *p* = .225 for NPI score) or NfL (β = 0.094, *p* = .659 for MMSE scores; β = −0.104, *p* = .242 for NPI score) levels in SCGs (Table 3). An exploratory analysis of the NPI subdomains revealed that greater changes in hallucination scores of care recipients were associated with an increase in NfL levels among SCGs (β = 0.431, *p* = .032). However, after applying the Bonferroni correction, the result was not significant (Supplementary Table 2).

**Table 3.** Longitudinal Association of Caregiving Burden With GFAP and NfL Levels Among SCGs

	Δ GFAP			Δ NfL		
	β	*t*	*p* Value	β	*t*	*p* Value
Model 1*						
Δ GDS of SCG	0.609	3.608	0.001	0.194	0.954	0.348
Δ MMSE of care recipient	−0.148	−0.839	0.408	0.037	0.105	0.917
Δ NPI	−0.378	−1.013	0.319	−0.123	−1.434	0.161
Model 2†						
Δ GDS of SCG	0.763	3.374	0.003	0.392	1.562	0.130
Δ MMSE of care recipient	0.087	0.383	0.705	0.094	0.447	0.659
Δ NPI	−0.188	−1.241	0.225	−0.104	−1.194	0.242

*Notes*: GDS = Geriatric Depression Scale; GFAP = glial fibrillary acidic protein; MMSE = Mini-Mental State Examination; NfL = neurofilament light chain; NPI = neuropsychiatric inventory; SCG = spousal caregiver.

*Model 1: Adjusted for age and sex of SCGs.

†Model 2: Adjusted for age, sex, APOE4 positivity, VRS, interval, IPAQ scores, and baseline MMSE of care recipients or baseline GDS of SCGs (and BMI for GFAP analyses).

### Interaction Effect of Sex, APOE4 Status, and Clinical Diagnosis of Care Recipients

We also tested whether the influence of changes in depression on the changes in GFAP levels differed according to sex, APOE4 status, or clinical diagnosis of the care recipient. However, none of the variables showed significant interaction effects (Table 4).

**Table 4.** Moderating Effects of Sex, APOE4 Positivity of SCGs, and Clinical Diagnosis of Care Recipients on Associations Between Changes in GDS and GFAP Among SCGs

Variable	β	*t*	*p* Value
Δ GFAP			
Model for sex effect			
Δ GDS	0.554	1.597	0.123
Sex	0.088	0.446	0.660
Δ GDS × Sex	0.134	0.290	0.774
Model for APOE4 positivity effect			
Δ GDS	0.550	2.063	0.005
APOE4 positivity	0.143	0.267	0.792
Δ GDS × APOE4	0.274	0.642	0.527
Model for clinical diagnosis of care recipient effect			
Δ GDS	0.689	1.859	0.075
Clinical diagnosis*	−0.967	−2.262	0.033
Δ GDS × clinical diagnosis	−0.075	−0.185	0.855

*Notes*: The multiple logistic regression model included Δ GDS, sex (or APOE4 or clinical diagnosis), and the interaction between Δ GDS and sex (or APOE4 or clinical diagnosis), all treated as independent variables. Age, sex, APOE4 positivity, VRS, IPAQ scores, follow-up intervals, BMI, and baseline GDS were treated as covariates when appropriate, and annualized △ GFAP was treated as the dependent variable. APOE = apolipoprotein E; BMI = body mass index; GDS = Geriatric Depression Scale; GFAP = glial fibrillary acidic protein; IPAQ = International Physical Activity Questionnaire; SCG = spousal caregiver.

*Cognitively normal versus mild cognitive impairment.

### Association of Plasma GFAP and NfL Levels With Cognition in SCGs

The association between plasma biomarkers and the cognitive function was also examined among SCGs. No significant relationship was found between baseline levels of GFAP (β = 0.305, *p* = .070) and NfL (β = −0.254, *p* = .167) and MMSE scores in SCGs. Similarly, there was no significant with changes in MMSE scores (β = −0.136, *p* = .455 for GFAP and β = 0.204, *p* = .310 for NfL). Furthermore, longitudinal analyses revealed that changes in GFAP levels (β = 0.110, *p* = .546) and NfL levels (β = −0.138, *p* = .418) were not significantly related to changes in MMSE scores (Supplementary Table 3).

## Discussion

In this longitudinal study, we aimed to explore the impact of caregiving burden on neuroinflammation and neurodegeneration among SCGs to individuals with cognitive decline. The results showed a significant correlation between increased depressive symptoms in SCGs and elevated levels of GFAP, a biomarker of neuroinflammation. These results provide novel insights into the influence of caregiving to patients with cognitive decline on the neurobiological alterations of the SCGs, specifically regarding GFAP and NfL levels. Our fingdings highlight potential neurobiological mechanisms underlying the health risks faced by SCGs of older adults with cognitive impairment.

Previous studies have primarily focused on the psychological and social consequences of caregiving to patients with cognitive impairment (1,3,6) and documented increased risks of dementia (13) and cardiovascular disease (11,12,15) among caregivers. Our study adds a neurobiological evidence by demonstrating a correlation between increased depressive symptoms and GFAP levels, even after adjusting for VRS, BMI, IPAQ scores, and APOE4 status, which could affect GFAP levels. This supports the hypothesis that neuroinflammation mediates the relationship between caregiving-related stress and its health consequences, aligning with prior evidence linking depressive symptoms to neuroinflammatory responses (43–46).

Research has shown increased GFAP levels in the cerebrospinal fluid (46) and blood (44) of patients with depression, indicating astrocytic activation (27), which is a feature of depression (47). Astroglial proteins, such as GFAP, have already been shown to be candidate markers for neurodegenerative diseases (25,48). Recent large-scale longitudinal studies have shown that elevated GFAP levels are robust prognostic markers for all-cause dementia (25,29–31). Supporting our findings, the study by Vitaliano et al. (20) demonstrated that increased CRP levels over a 2-year period were associated with functional decline in caregivers to individuals with dementia, compared to demographically matched non-caregivers even after adjusting for baseline functional impairment and psychological distress. These findings suggest that caregiving and associated inflammation play key roles in the accelerated functional decline observed in caregivers. While Vitaliano et al.’s work focuses on systemic inflammation (20), our study extends this to neuroinflammation, as reflected in elevated GFAP levels. Additionally, previous research has demonstrated that compared with controls, the SCGs to individuals with dementia have a 1.6-fold increased risk of dementia incidence over a 14-year longitudinal follow-up period (13). Integrating these previous findings with the results of our study suggests that neuroinflammation, potentially induced by depression, plays a significant role in mediating adverse functional and neurocognitive outcomes in SCGs.

Specific normative data for GFAP and NfL levels in the Korean population have yet to be established. However, the baseline levels in our participants (188.2 ± 76.0 pg/mL for GFAP and 21.6 ± 12.3 pg/mL for NfL) were within the normative ranges reported for a similarly aged Canadian cohort (40.7–228 pg/mL for GFAP and 8.1–47.1 pg/mL for NfL in individuals aged 60–80) (49). Given the emerging evidence linking GFAP and NfL levels to dementia prediction in healthy older adults (25,26), the increasing depressive symptoms reported by SCGs to spouses without dementia need close attention. This observation suggests the potential for early detection and intervention for dementia, emphasizing the critical role of monitoring depressive symptoms in SCGs as indicators of underlying neuroinflammation and an increased risk of neurodegeneration.

The finding that NfL levels were not correlated with GDS scores at baseline, as well as the lack of an association between their longitudinal changes, aligns with previous findings (44,50) that indicated no significant differences in NfL levels between patients with depression and healthy controls. This suggests a more direct involvement of caregiving-related stress in neuroinflammatory processes than in neurodegenerative changes, as indicated by NfL levels. In our study, changes in GDS scores were not associated with changes in NfL levels; however, a positive correlation was observed with follow-up NfL levels (data not shown). Therefore, further longitudinal studies are needed to determine whether the neuroinflammation caused by stress affects neurodegeneration in caregivers.

There was no significant association between the baseline and longitudinal changes in the MMSE scores of SCGs and plasma biomarkers. One possible explanation for this finding is that the SCGs in our cohort, who were capable of accompanying their spouses with cognitive decline to a university hospital for treatment, may already possess resilience. Additionally, the follow-up period of 16 months may have been too short to observe significant relationships with longitudinal MMSE changes. Previous studies that have identified GFAP and NfL levels as prognostic factors for dementia typically involved much longer follow-up durations, ranging from 4 to 14 years (25,26,29).

The results of our study indicated no relationship between the degree of cognitive impairment or neuropsychiatric symptoms in care recipients and plasma biomarkers, either cross-sectionally or longitudinally. This suggests that depression experienced by SCGs may have a more significant impact on neuroinflammation than the severity of the care recipient’s cognitive impairment or neuropsychiatric symptoms. Moreover, our analysis did not reveal significant interaction effects between changes in depressive symptoms and GFAP or NfL levels across sex, APOE4 status, or clinical diagnosis of care recipients. This finding indicates that caregiving-induced psychological distress is a pervasive risk factor for neuroinflammation, independent of sex, APOE4 status, or the severity of cognitive impairment in care recipients.

These findings have significant clinical implications. SCGs are often referred to as the “invisible second patient,” as they are known to have a higher risk of not only dementia but also all-cause mortality (17). GFAP is a strong prognostic blood-based biomarker of the incidence of dementia in older adults (25,29,32). The significant association between depressive symptoms and increased GFAP levels emphasizes the need for clinicians to actively monitor SCGs for signs of depression. Early identification and intervention can mitigate the risk of cognitive impairment, emphasizing the importance of supporting mental health and well-being in SCGs.

Despite the strengths of our study, it has several limitations. First, the recruitment of participants from a tertiary university hospital, all of whom were healthy enough to provide care to spouses with cognitive decline, introduced a potential selection bias. This bias may skew the results toward the null hypothesis, suggesting that the impact of depression on neuroinflammation may be underestimated in SCGs. Second, the recruitment period coincided with the COVID-19 pandemic, which necessitates caution when interpreting these results. In Korea, strict isolation measures were implemented early on for patients with confirmed COVID-19 and their contacts. During the recruitment period, individuals with respiratory symptoms or a temperature above 37.5°C were restricted from entering hospitals. Therefore, the impact of infection on participant evaluation is believed to have been minimal. Third, our study did not use the zarit burden interview, a commonly used measure of caregiving burden. Instead, we assessed cognitive decline and neuropsychiatric symptoms of dementia in care recipients and perceived stress levels in caregivers. This approach provided a more comprehensive view of the factors influencing neuroinflammation and neurodegeneration in SCGs. By considering multiple aspects of caregiving and using measures routinely assessed in clinical settings, our method may have advantages in terms of applicability and feasibility in real-world clinical use. Lastly, although this study employed a longitudinal design, it had a relatively small sample size. Nevertheless, we identified significant associations between depressive symptoms in SCGs and markers of neuroinflammation, even after adjusting for various factors known to influence neuroinflammatory processes, including VRS, physical activity levels and BMI. Further research involving larger cohorts and extended follow-up periods is warranted to validate and expand our observations.

In conclusion, this study contributes to the understanding of neuroinflammatory responses to caregiving-related stress, providing a crucial link between the psychological burden of caregiving and its neurobiological consequences. Future studies should focus on delineating the specific molecular pathways involved and investigating targeted interventions to alleviate neuroinflammatory responses in SCGs. Addressing these challenges is crucial for developing comprehensive strategies to support the health and well-being of SCGs, who are themselves older adults and at risk of neurodegenerative disorders.

## Supplementary Material

glae235_suppl_Supplementary_Tables_S1-S3

## Data Availability

The data sets generated and analyzed during the present study are not publicly available due to ethical considerations and privacy restrictions. However, the data can be made available by the corresponding author upon request, subject to approval by the Institutional Review Board of the Chungnam National University Hospital, South Korea.

## References

[1] Vitaliano PP , Schulz R, Kiecolt-Glaser J, Grant I. Research on physiological and physical concomitants of caregiving: where do we go from here? Ann Behav Med. 1997;19(2):117–123. https://doi.org/10.1007/BF02883328 9603686

[2] Schulz R , Beach SR, Czaja SJ, Martire LM, Monin JK. Family caregiving for older adults. Annu Rev Psychol. 2020;71:635–659. https://doi.org/10.1146/annurev-psych-010419-050754 31905111 PMC7291827

[3] Vitaliano PP , Zhang J, Scanlan JM. Is caregiving hazardous to one’s physical health? A meta-analysis. Psychol Bull. 2003;129(6):946–972. https://doi.org/10.1037/0033-2909.129.6.946 14599289

[4] Schulz R , Sherwood PR. Physical and mental health effects of family caregiving. J Soc Work Educ. 2008;44(Suppl):105–113. https://doi.org/10.5175/jswe.2008.773247702 PMC2791523 18797217

[5] Vitaliano PP , Murphy M, Young HM, Echeverria D, Borson S. Does caring for a spouse with dementia promote cognitive decline? A hypothesis and proposed mechanisms. J Am Geriatr Soc. 2011;59(5):900–908. https://doi.org/10.1111/j.1532-5415.2011.03368.x 21568959

[6] Snyder SA , Vitaliano PP. Caregiver psychological distress: longitudinal relationships with physical activity and diet. Am J Alzheimers Dis Other Demen. 2020;35:1533317520904554. https://doi.org/10.1177/1533317520904554 32066252 PMC10624019

[7] Beeson RA. Loneliness and depression in spousal caregivers of those with Alzheimer’s disease versus non-caregiving spouses. Arch Psychiatr Nurs. 2003;17(3):135–143. https://doi.org/10.1016/s0883-9417(03)00057-8 12840806

[8] Joling KJ , van Hout HPJ, Schellevis FG, et al Incidence of depression and anxiety in the spouses of patients with dementia: a naturalistic cohort study of recorded morbidity with a 6-year follow-up. Am J Geriatr Psychiatry. 2010;18(2):146–153. https://doi.org/10.1097/JGP.0b013e3181bf9f0f 20104070

[9] Ory MG , Hoffman RR III, Yee JL, Tennstedt S, Schulz R. Prevalence and impact of caregiving: a detailed comparison between dementia and nondementia caregivers. Gerontologist. 1999;39(2):177–185. https://doi.org/10.1093/geront/39.2.177 10224714

[10] Aggarwal B , Liao M, Christian A, Mosca L. Influence of caregiving on lifestyle and psychosocial risk factors among family members of patients hospitalized with cardiovascular disease. J Gen Intern Med. 2009;24(1):93–98. https://doi.org/10.1007/s11606-008-0852-1 18998190 PMC2607516

[11] Xu XY , Kwan RYC, Leung AYMJ. Factors associated with the risk of cardiovascular disease in family caregivers of people with dementia: a systematic review. J Int Med Res. 2020;48(1):300060519845472. https://doi.org/10.1177/0300060519845472 31115265 PMC7140198

[12] Von Känel R , Mausbach BT, Patterson TL, et al Increased Framingham Coronary Heart Disease Risk Score in dementia caregivers relative to non-caregiving controls. Gerontology. 2008;54(3):131–137. https://doi.org/10.1159/000113649 18204247

[13] Norton MC , Smith KR, Østbye T, et al; Cache County Investigators. Greater risk of dementia when spouse has dementia? The Cache County Study. J Am Geriatr Soc. 2010;58(5):895–900. https://doi.org/10.1111/j.1532-5415.2010.02806.x 20722820 PMC2945313

[14] Dassel KB , Carr DC, Vitaliano P. Does caring for a spouse with dementia accelerate cognitive decline? Findings from the Health and Retirement Study. Gerontologist. 2017;57(2):319–328. https://doi.org/10.1093/geront/gnv148 26582383

[15] Mausbach BT , Patterson TL, Rabinowitz YG, Grant I, Schulz R. Depression and distress predict time to cardiovascular disease in dementia caregivers. Health Psychol. 2007;26(5):539–544. https://doi.org/10.1037/0278-6133.26.5.539 17845105

[16] Wolff JL , Mulcahy J, Huang J, Roth DL, Covinsky K, Kasper JD. Family caregivers of older adults, 1999-2015: trends in characteristics, circumstances, and role-related appraisal. Gerontologist. 2018;58(6):1021–1032. https://doi.org/10.1093/geront/gnx093 28637266 PMC6215459

[17] Schulz R , Beach SR. Caregiving as a risk factor for mortality: the Caregiver Health Effects Study. JAMA. 1999;282(23):2215–2219. https://doi.org/10.1001/jama.282.23.2215 10605972

[18] Potier F , Degryse J-M, de Saint-Hubert M. Impact of caregiving for older people and pro-inflammatory biomarkers among caregivers: a systematic review. Aging Clin Exp Res. 2018;30(2):119–132. https://doi.org/10.1007/s40520-017-0765-0 28474314

[19] Gouin J-P , Glaser R, Malarkey WB, Beversdorf D, Kiecolt-Glaser J. Chronic stress, daily stressors, and circulating inflammatory markers. Health Psychol. 2012;31(2):264–268. https://doi.org/10.1037/a0025536 21928900 PMC3253267

[20] Vitaliano P , Echeverria D, Shelkey M, Zhang J, Scanlan J. A cognitive psychophysiological model to predict functional decline in chronically stressed older adults. J Clin Psychol Med Settings. 2007;14(3):177–190. https://doi.org/10.1007/s10880-007-9071-x

[21] Kaptoge S , Seshasai SRK, Gao P, et al Inflammatory cytokines and risk of coronary heart disease: new prospective study and updated meta-analysis. Eur Heart J. 2014;35(9):578–589. https://doi.org/10.1093/eurheartj/eht367 24026779 PMC3938862

[22] Darweesh SKL , Wolters FJ, Ikram MA, de Wolf F, Bos D, Hofman A. Inflammatory markers and the risk of dementia and Alzheimer’s disease: a meta-analysis. Alzheimers Dement. 2018;14(11):1450–1459. https://doi.org/10.1016/j.jalz.2018.02.014 29605221

[23] Koyama A , O’Brien J, Weuve J, Blacker D, Metti AL, Yaffe K. The role of peripheral inflammatory markers in dementia and Alzheimer’s disease: a meta-analysis. J Gerontol A Biol Sci Med Sci. 2013;68(4):433–440. https://doi.org/10.1093/gerona/gls187 22982688 PMC3693673

[24] Troubat R , Barone P, Leman S, et al Neuroinflammation and depression: a review. Eur J Neurosci. 2021;53(1):151–171. https://doi.org/10.1111/ejn.14720 32150310

[25] Guo Y , You J, Zhang Y, et al Plasma proteomic profiles predict future dementia in healthy adults. Nat Aging. 2024;4(2):247–260. https://doi.org/10.1038/s43587-023-00565-0 38347190

[26] Verberk IMW , Laarhuis MB, van den Bosch KA, et al Serum markers glial fibrillary acidic protein and neurofilament light for prognosis and monitoring in cognitively normal older people: a prospective memory clinic-based cohort study. Lancet Healthy Longev. 2021;2(2):e87–e95. https://doi.org/10.1016/S2666-7568(20)30061-1 36098162

[27] Pekny M , Nilsson M. Astrocyte activation and reactive gliosis. Glia. 2005;50(4):427–434. https://doi.org/10.1002/glia.20207 15846805

[28] Bridel C , Van Wieringen WN, Zetterberg H, et al; and the NFL Group. Diagnostic value of cerebrospinal fluid neurofilament light protein in neurology: a systematic review and meta-analysis. JAMA Neurol. 2019;76(9):1035–1048. https://doi.org/10.1001/jamaneurol.2019.1534 31206160 PMC6580449

[29] Dark HE , Paterson C, Daya GN, et al Proteomic indicators of health predict Alzheimer’s disease biomarker levels and dementia risk. Ann Neurol. 2024;95(2):260–273. https://doi.org/10.1002/ana.26817 37801487 PMC10842994

[30] Cicognola C , Janelidze S, Hertze J, et al Plasma glial fibrillary acidic protein detects Alzheimer pathology and predicts future conversion to Alzheimer dementia in patients with mild cognitive impairment. Alzheimers Res Ther. 2021;13(1):68. https://doi.org/10.1186/s13195-021-00804-9 33773595 PMC8005231

[31] Tang Y , Han L, Li S, et al Plasma GFAP in Parkinson’s disease with cognitive impairment and its potential to predict conversion to dementia. npj Parkinson's Dis. 2023;9(1):23. https://doi.org/10.1038/s41531-023-00447-7 36759508 PMC9911758

[32] Cronjé HT , Liu X, Odden MC, et al Serum NfL and GFAP are associated with incident dementia and dementia mortality in older adults: the Cardiovascular Health Study. Alzheimers Dement. 2023;19(12):5672–5680. https://doi.org/10.1002/alz.13367 37392405 PMC10757989

[33] Dauphinot V , Delphin-Combe F, Mouchoux C, et al Risk factors of caregiver burden among patients with Alzheimer’s disease or related disorders: a cross-sectional study. J Alzheimers Dis. 2015;44(3):907–916. https://doi.org/10.3233/JAD-142337 25374109

[34] Black CM , Ritchie CW, Khandker RK, et al Non-professional caregiver burden is associated with the severity of patients’ cognitive impairment. PLoS One. 2018;13(12):e0204110. https://doi.org/10.1371/journal.pone.0204110 30521532 PMC6283568

[35] Jeon SY , Kim JL. Caregiving for a spouse with cognitive impairment: effects on nutrition and other lifestyle factors. J Alzheimers Dis. 2021;84(3):995–1003. https://doi.org/10.3233/JAD-210694 34602480

[36] Lee DY , Lee KU, Lee JH, et al A normative study of the Mini-Mental State Examination in the Korean elderly. J Korean Neuropsychiatr Assoc. 2002;41:508–525.

[37] Bae JN , Cho MJ. Development of the Korean version of the Geriatric Depression Scale and its short form among elderly psychiatric patients. J Psychosom Res. 2004;57(3):297–305. https://doi.org/10.1016/j.jpsychores.2004.01.004 15507257

[38] Cummings JL , Mega M, Gray K, Rosenberg-Thompson S, Carusi DA, Gornbein J. The Neuropsychiatric Inventory: comprehensive assessment of psychopathology in dementia. Neurology. 1994;44(12):2308–2314. https://doi.org/10.1212/wnl.44.12.2308 7991117

[39] DeCarli C , Mungas D, Harvey D, et al Memory impairment, but not cerebrovascular disease, predicts progression of MCI to dementia. Neurology. 2004;63(2):220–227. https://doi.org/10.1212/01.wnl.0000130531.90205.ef 15277612 PMC1820872

[40] Chun MY. Validity and reliability of Korean version of International Physical Activity Questionnaire short form in the elderly. Korean J Fam Med. 2012;33(3):144–151. https://doi.org/10.4082/kjfm.2012.33.3.144 22787536 PMC3391639

[41] Ainsworth BE , Haskell WL, Whitt MC, et al Compendium of physical activities: an update of activity codes and MET intensities. Med Sci Sports Exerc. 2000;32(9 Suppl):S498–S504. https://doi.org/10.1097/00005768-200009001-00009 10993420

[42] Wenham PR , Price WH, Blandell G. Apolipoprotein E genotyping by one-stage PCR. Lancet. 1991;337(8750):1158–1159. https://doi.org/10.1016/0140-6736(91)92823-k 1674030

[43] Schroeter ML , Sacher J, Steiner J, Schoenknecht P, Mueller K. Serum S100B represents a new biomarker for mood disorders. Curr Drug Targets. 2013;14(11):1237–1248. https://doi.org/10.2174/13894501113149990014 23701298 PMC3821390

[44] Steinacker P , Al Shweiki MR, Oeckl P, et al Glial fibrillary acidic protein as blood biomarker for differential diagnosis and severity of major depressive disorder. J Psychiatr Res. 2021;144:54–58. https://doi.org/10.1016/j.jpsychires.2021.09.012 34600287

[45] Güleş E , Iosifescu DV, Tural U. Plasma neuronal and glial markers and anterior cingulate metabolite levels in major depressive disorder: a pilot study. Neuropsychobiology. 2020;79(3):214–221. https://doi.org/10.1159/000505782 32045918

[46] Michel M , Fiebich BL, Kuzior H, et al Increased GFAP concentrations in the cerebrospinal fluid of patients with unipolar depression. Transl Psychiatry. 2021;11(1):308. https://doi.org/10.1038/s41398-021-01423-6 34021122 PMC8139962

[47] Wang Q , Jie W, Liu JH, Yang JM, Gao TM. An astroglial basis of major depressive disorder? An overview. Glia. 2017;65(8):1227–1250. https://doi.org/10.1002/glia.23143 28317185

[48] Oeckl P , Halbgebauer S, Anderl-Straub S, et al; Consortium for Frontotemporal Lobar Degeneration German. Glial fibrillary acidic protein in serum is increased in Alzheimer’s disease and correlates with cognitive impairment. J Alzheimers Dis. 2019;67(2):481–488. https://doi.org/10.3233/JAD-180325 30594925

[49] Cooper JG , Stukas S, Ghodsi M, et al Age specific reference intervals for plasma biomarkers of neurodegeneration and neurotrauma in a Canadian population. Clin Biochem. 2023;121-122:110680. https://doi.org/10.1016/j.clinbiochem.2023.110680 37884086

[50] Al Shweiki MR , Steinacker P, Oeckl P, et al Neurofilament light chain as a blood biomarker to differentiate psychiatric disorders from behavioural variant frontotemporal dementia. J Psychiatr Res. 2019;113:137–140. https://doi.org/10.1016/j.jpsychires.2019.03.019 30953863

