# Supplementary material for: Caregiving-Related Depression Increases Neuroinflammation in Spousal Caregivers to Individuals With Cognitive Impairment: A Longitudinal Study
Source: J Gerontol A Biol Sci Med Sci. 2024 Sep 19;79(11):glae235. doi: 10.1093/gerona/glae235 (PMC11638088; doi:10.1093/gerona/glae235)
Supplement: glae235_suppl_Supplementary_Tables_S1-S3 [file glae235_suppl_supplementary_tables_s1-s3.docx]

**eTable 1. Baseline association of GFAP and NfL levels with care-recipient NPI subdomains**

|  | **Baseline GFAP** | | | | **Baseline NfL** | | | |
| --- | --- | --- | --- | --- | --- | --- | --- | --- |
| **Each NPI subdomains** | ***β*** | ***t*** | ***p-value^a^*** | ***p-value^b^*** | ***β*** | ***t*** | ***p-value^a^*** | ***p-value^b^*** |
| Baseline NPI_delusions | -0.075 | -0.444 | 0.660 | 0.491 | -0.038 | -0.240 | 0.812 | 0.560 |
| Baseline NPI_hallucinations | 0.067 | 0.387 | 0.701 | 0.144 | -0.052 | -0.320 | 0.751 | 0.532 |
| Baseline NPI_agitation/aggression | 0.016 | 0.091 | 0.928 | 0.872 | -0.002 | -0.125 | 0.902 | 0.890 |
| Baseline NPI_depression/dysphoria | 0.039 | 0.232 | 0.818 | 0.990 | -0.042 | -0.267 | 0.791 | 0.674 |
| Baseline NPI_anxiety | -0.079 | -0.467 | 0.644 | 0.608 | 0.106 | 0.668 | 0.509 | 0.602 |
| Baseline NPI_elation/euphoria | -0.059 | -0.346 | 0.732 | 0.740 | -0.166 | -1.056 | 0.299 | 0.296 |
| Baseline NPI_apathy/indifference | -0.019 | -0.111 | 0.913 | -0.041 | 0.178 | 1.107 | 0.277 | 0.337 |
| Baseline NPI_disinhibition | -0.149 | -0.869 | 0.391 | 0.249 | -0.132 | -0.818 | 0.419 | 0.596 |
| Baseline NPI_irritability/lability | -0.056 | -0.332 | 0.742 | 0.566 | -0.096 | -0.607 | 0.548 | 0.563 |
| Baseline NPI_aberrant motor behavior | -0.144 | -0.852 | 0.401 | 0.210 | -0.064 | -0.401 | 0.691 | 0.811 |
| Baseline NPI_nighttime disturbnces | -0.182 | -1.051 | 0.301 | 0.340 | -0.207 | -1.285 | 0.208 | 0.255 |
| Baseline NPI_changes in appetite | -0.012 | -0.068 | 0.946 | 0.796 | 0.004 | 0.025 | 0.980 | 0.928 |

*Note.* GFAP: glial fibrillary acidic protein; NfL: neurofilament-light-chain; NPI: Neuropsychiatric Inventory; MMSE: Mini-Mental State Examination; APOE: apolipoprotein E; VRS: vascular risk factor score; IPAQ: International Physical Activity Questionnaire; SCG: spouse caregiver; BMI: body mass index

^a^Adjusted for age and sex

^b^Adjusted for age, sex, APOE, and VRS, IPAQ of SCGs (and BMI for GFAP analyses)

**eTable 2. Longitudinal association of GFAP and NfL levels with care-recipient NPI subdomains**

|  | **Δ GFAP** | | | | **Δ NfL** | | | |
| --- | --- | --- | --- | --- | --- | --- | --- | --- |
|  | ***β*** | ***t*** | ***p-value^a^*** | ***p-value^b^*** | ***β*** | ***t*** | ***p-value^a^*** | ***p-value^b^*** |
| Δ NPI_delusions | 0.198 | 1.094 | 0.283 | 0.338 | -0.013 | -0.068 | 0.946 | 0.635 |
| Δ NPI_hallucinations | 0.235 | 1.220 | 0.232 | 0.249 | 0.383 | 2.093 | 0.045 | 0.032 |
| Δ NPI_agitation/aggression | 0.040 | 0.220 | 0.827 | 0.782 | -0.276 | -1.61 | 0.118 | 0.120 |
| Δ NPI_depression/dysphoria | -0.086 | -0.469 | 0.643 | 0.782 | 0.110 | 0.601 | 0.552 | 0.734 |
| Δ NPI_anxiety | 0.256 | 1.415 | 0.167 | 0.237 | -0.104 | -0.563 | 0.577 | 0.792 |
| Δ NPI_elation/euphoria | -0.186 | -1.031 | 0.311 | 0.299 | -0.134 | -0.739 | 0.465 | 0.646 |
| Δ NPI_apathy/indifference | 0.070 | 0.382 | 0.705 | 0.882 | -0.015 | -0.080 | 0.937 | 0.901 |
| Δ NPI_disinhibition | 0.062 | 0.341 | 0.736 | 0.516 | 0.058 | 0.319 | 0.752 | 0.638 |
| Δ NPI_irritability/lability | -0.056 | -0.308 | 0.760 | 0.825 | 0.154 | 0.859 | 0.397 | 0.535 |
| Δ NPI_aberrant motor behavior | 0.059 | 0.324 | 0.748 | 0.944 | 0.055 | 0.305 | 0.762 | 0.808 |
| Δ NPI_nighttime disturbances | -0.036 | -0.198 | 0.844 | 0.795 | -0.113 | -0.625 | 0.537 | 0.379 |
| Δ NPI_changes in appetite | -0.218 | -1.243 | 0.224 | 0.175 | -0.211 | -1.214 | 0.234 | 0.642 |

*Note.* GFAP: glial fibrillary acidic protein; NfL: neurofilament-light-chain; NPI: Neuropsychiatric Inventory; MMSE: Mini-Mental State Examination; APOE: apolipoprotein E; VRS: vascular risk factor score; IPAQ: International Physical Activity Questionnaire; SCG: spouse caregiver; BMI: body mass index

^a^Adjusted for age and sex

^b^Adjusted for age, sex, APOE, and VRS, interval, IPAQ scores of SCGs and Baseline levels of each NPI subdomain (and BMI for GFAP analyses)

**eTable 3. Associations between Baseline and longitudinal changes in GFAP and NfL levels and MMSE scores in SCGs**

| **Dependent variables** | **Independent variable** | ***β*** | ***t*** | ***p-value****^a^* |
| --- | --- | --- | --- | --- |
| Baseline MMSE of SCG | Baseline GFAP | 0.305 | 1.875 | 0.070 |
|  | Baseline NfL | -0.254 | -1.415 | 0.167 |
|  |  | ***β*** | ***t*** | ***p-value****^b^* |
| ΔMMSE of SCG | Baseline GFAP | -0.136 | -0.757 | 0.455 |
|  | Baseline NfL | 0.204 | 1.033 | 0.310 |
|  | Δ GFAP | 0.110 | 0.611 | 0.546 |
|  | Δ NfL | -0.138 | -0.824 | 0.418 |

*Note.* GFAP: glial fibrillary acidic protein; SCG: spouse caregiver; MMSE: Mini-Mental State Examination; APOE: apolipoprotein E.

^a^Adjusted for age, sex and APOE4.

^b^Adjusted for age, sex, APOE4 and intervals.
